# Supplementary material for: Modelling Skylarks (Alauda arvensis) to Predict Impacts of Changes in Land Management and Policy: Development and Testing of an Agent-Based Model
Source: PLoS One. 2013 Jun 6;8(6):e65803. doi: 10.1371/journal.pone.0065803 (PMC3675089; doi:10.1371/journal.pone.0065803)
Supplement: Supporting Information S4 — The skylark ODdox as a zipped archive. (ZIP) [file pone.0065803.s004.zip › Skylark_ODdox/rastermap_8h.html]

ALMaSS Skylark ODdox: rastermap.h File Reference


ALMaSS Skylark ODdox
 2.0


- Main Page
- Related Pages
- Classes
- Files

- File List
- File Members

Classes |
Variables

rastermap.h File Reference

`#include <cstdlib>`

|  |  |
| --- | --- |
| Classes | |
| class | RasterMap |

|  |  |
| --- | --- |
| Variables | |
| int \* | m\_polymapping |

---

## Variable Documentation

int\* m\_polymapping

Referenced by Landscape::BeetleBankAdd(), Landscape::BorderAdd(), Landscape::BorderScan(), Landscape::BorderScan2(), Landscape::ChangeMapMapping(), Landscape::hb\_Add(), Landscape::hb\_GenerateHBPolys(), Landscape::OrchardBorderAdd(), Landscape::PolysRemoveInvalid(), Landscape::PolysValidate(), RasterMap::RasterMap(), Landscape::ReadPolys(), Landscape::SetLESignal(), Landscape::SupplyDayDegrees(), Landscape::SupplyDeadBiomass(), Landscape::SupplyElementSubType(), Landscape::SupplyElementType(), Landscape::SupplyFarmArea(), Landscape::SupplyFarmIntensity(), Landscape::SupplyFarmOwner(), Landscape::SupplyFarmOwnerIndex(), Landscape::SupplyFarmType(), Landscape::SupplyGrazingPressure(), Landscape::SupplyGreenBiomass(), Landscape::SupplyHasTramlines(), Landscape::SupplyInsects(), Landscape::SupplyIsGrass(), Landscape::SupplyJustMown(), Landscape::SupplyJustSprayed(), Landscape::SupplyLAGreen(), Landscape::SupplyLastTreatment(), Landscape::SupplyLEPointer(), Landscape::SupplyLESignal(), Landscape::SupplyPesticideCell(), Landscape::SupplySkScrapes(), Landscape::SupplyTrafficLoad(), Landscape::SupplyValidX(), Landscape::SupplyValidY(), Landscape::SupplyVegAge(), Landscape::SupplyVegBiomass(), Landscape::SupplyVegCover(), Landscape::SupplyVegDensity(), Landscape::SupplyVegDigestability(), Landscape::SupplyVegHeight(), Landscape::SupplyVegPatchy(), Landscape::SupplyVegType(), Landscape::SupplyWeedBiomass(), Landscape::UnsprayedMarginAdd(), Landscape::UnsprayedMarginScan(), and Landscape::~Landscape().


- CJT
- MSVC
- ALMaSS Working Source
- Landscape
- rastermap.h
- Generated on Thu Jan 10 2013 13:15:35 for ALMaSS Skylark ODdox by
   1.8.1.1
